# Precision monitoring of rumination and locomotion in relation to milk fat-to-protein ratio in early lactation dairy cattle

**DOI:** 10.3389/fvets.2025.1632224

**Published:** 2025-07-09

**Authors:** Samanta Arlauskaitė, Akvilė Girdauskaitė, Arūnas Rutkauskas, Karina Džermeikaitė, Justina Krištolaitytė, Mindaugas Televičius, Dovilė Malašauskienė, Lina Anskienė, Sigitas Japertas, Walter Baumgartner, Ramūnas Antanaitis

**Affiliations:** ^1^Large Animal Clinic, Veterinary Academy, Lithuanian University of Health Sciences, Kaunas, Lithuania; ^2^Department of Animal Breeding, Faculty of Animal Sciences, Lithuanian University of Health Sciences, Kaunas, Lithuania; ^3^Practical Training and Research Center, Lithuanian University of Health Sciences, Kaunas, Lithuania; ^4^Clinical Centre for Ruminant and Camelid Medicine, University of Veterinary Medicine, Vienna, Austria

**Keywords:** dairy cows, innovative technologies, fat-to-protein ratio, rumination, locomotion

## Abstract

The milk fat-to-protein ratio (FPR) is a valuable indicator of metabolic health in dairy cows, especially during early lactation when cows are most susceptible to negative energy balance. This study aimed to evaluate the relationship between FPR, milk composition, blood biochemical parameters, and behavioral indicators in early-lactation Holstein cows. Twenty-seven cows between 9 and 59 days in milk were monitored and categorized into three groups: low-grade ruminal acidosis (LGRA; FPR < 1.2), healthy (H; FPR 1.2–1.5), and subclinical ketosis (SCK; FPR > 1.5). Milk composition was assessed in real time using the Brolis HerdLine in-line analyzer, while rumination time, reticulorumen temperature, water intake, and activity were recorded using SmaXtec boluses. Blood samples were collected weekly to analyze metabolic and biochemical parameters. Cows in the SCK group exhibited significantly lower milk lactose and protein concentrations, shorter rumination time, lower iron levels, and higher milk fat content, NEFA concentrations, and activity levels compared to the LGRA and healthy groups. The study demonstrated that elevated FPR is associated with metabolic and behavioral changes indicative of subclinical metabolic disorders, particularly subclinical ketosis. The integration of real-time milk composition data, behavioral monitoring, and blood biochemical analysis enables a comprehensive and non-invasive approach for early detection and management of metabolic imbalances in dairy herds. This study highlights the potential of precision monitoring technologies to improve animal welfare and productivity by supporting proactive herd health management.

## 1 Introduction

Rising world population is leading to a continuous increase in milk demand. Developed countries consume more dairy products than developing nations. To keep up with this growing demand, improved technological approaches are essential for boosting milk yield (1). Advancements in information technology have enabled the use of sensors to monitor cattle behavior, allowing for automatic and continuous observation. This approach is more efficient and less invasive compared to traditional manual monitoring methods (2, 3). Feeding behavior serves as a vital indicator of growth and health in cattle. Among these behaviors, rumination time and eating are the most direct and reliable traits for assessing the animal’s overall health condition (4). Milk composition in dairy cattle plays a vital role, particularly in terms of animal health, overall productivity, and the nutritional value of the milk (5). The Brolis HerdLine in-line milk analyzer (Brolis Sensor Technology, Vilnius, Lithuania) is an advanced spectroscopic sensor that measures lactose content and fat-to-protein ratios in bovine milk. This compact, reagent-free device provides real-time, continuous monitoring of milk composition during milking and can be integrated into milking stalls or automated systems with minimal maintenance (5).

Milk composition is influenced by substances derived from both blood and feed (6). Understanding the connections among components in feed, blood, and milk is important for accurately assessing the health and productivity of dairy animals (5).

The FPR in dairy cows is a key indicator of their energy balance and overall health, especially during the period of negative energy balance (NEB) after calving (7). Cows exhibiting suboptimal body condition score, particularly lower values during early lactation, were at increased risk of premature culling (8). The milk fat-to-protein ratio (FPR), calculated as the quotient of milk fat concentration to milk protein concentration, reflects the cow’s physiological status by integrating signals from both milk protein synthesis and body fat mobilization. This makes it a valuable tool for assessing the animal’s energy reserves available for maintenance and milk production (9). Cattle ketosis is a metabolic condition that adversely affects milk production and its components, notably influencing the fat-to-protein ratio. Elevated levels of ketone bodies, particularly *β*-hydroxybutyric acid (BHB), serve as a primary indicator of the disorder. In cases of subclinical ketosis (SCK), the FPR in milk frequently exceeds 1.4:1, reflecting the metabolic disturbances present in the cow (10). FPR exceeding 1.38 is associated with a 2.1-fold increase in the risk of clinical ketosis in cows. When the FPR surpasses 1.5, the likelihood of developing ketosis becomes even higher (11). Ketosis in cows most commonly occurs within the first two months of lactation (10). It was found that the FPR can be used as a non-invasive marker of metabolic health, especially in relation to acidosis. Their study showed that a higher FPR was associated with a reduction in milk yield by 4.97 kg/day. This effect was mostly pronounced between 5 and 30 days in milk (DIM), during which the average FPR was 1.32 ± 0.30 (12).

The connection between acidosis, ketosis, and rumination time in dairy cows is well-documented in the literature. Both SCK and subclinical acidosis (SCA) have been found to negatively influence rumination time. Specifically, cows with SCK showed a significant 17.47% decrease in rumination time, along with changes in their eating and chewing behaviors, highlighting the considerable impact of SCK on these vital activities (12). Ketosis is closely linked to reduced activity in dairy cows, especially during the transition period when they are most prone to metabolic issues. SCK often leads to lower activity levels, which can decline up to five days before clinical signs appear. That makes activity monitoring a useful early indicator of health problems (13). SCA impacts cow activity, with affected cows showing a significant decrease in walking time and other activity-related measures (12, 14). This study hypothesized that the milk FPR is significantly associated with changes in blood biochemical parameters, milk composition, and behavior in early-lactation cows, and may serve as an early marker of subclinical metabolic disorders. The aim was to evaluate the relationship between FPR and selected blood biomarkers, milk traits, and behavioral indicators using real-time data from in-line milk analyzers and intraruminal sensors.

## 2 Materials and methods

### 2.1 Animal housing conditions of this study

The study was conducted on a Lithuanian university of health sciences Practical Training and Research Center and large animal clinic, located in the central part of Lithuania in the eastern region of Europe. The attempt started on February 7th, 2025, and lasted until February 27th, 2025. Holstein dairy cows were selected for research. For this study, out of a total of 109 Holstein cows, we selected 27 (16 primiparous and 11 multiparous) cows for detailed monitoring. These cows were between 9 and 59 days in milk (DIM), with an average of 28 DIM. The animals were kept in a loose housing system and were fed a total mixed ration (TMR) year-round, formulated to meet their physiological needs. Lactating cows received a TMR consisting of approximately 31% corn silage, 10% grass silage, 4% grass hay, 49% grain concentrate mash, and 6% mineral mixture on a dry matter basis. The diet was designed to meet or exceed the nutritional requirements of a 550 kg Holstein cow producing 35 kg of milk per day. The ration contained 50.7% dry matter, 28.3% neutral detergent fiber, 19.8% acid detergent fiber, 38.7% non-fiber carbohydrates, 15.8% crude protein, and provided 1.60 Mcal/kg of net energy for lactation. Feeding was carried out daily at 08:00 and 16:00. The cows were milked using DeLaval milking robots (DeLaval Inc., Tumba, Sweden). The average body weight of the cows was 550 ± 45 kg. They were housed in ventilated free-stall barns. In 2024, the average energy-corrected milk yield (4.1% fat, 3.4% protein) was 10,304 kg per cow per year. Throughout the study, contact with the animals was kept to a minimum to ensure high standards of animal welfare.

### 2.2 Registration of parameters

In this study, milk composition was recorded using The Brolis HerdLine in-line milk analyzer (Brolis Sensor Technology, Vilnius, Lithuania), while rumination time, water intake, reticulorumen temperature and cow activity behaviors were monitored with the SmaXtec (SmaXtec animal care GmbH, Graz, Austria). Blood samples were collected once a week for five consecutive weeks, from the coccygeal vein at consistent time points on February 7, 12, 17, 22, and 27 in 2025.

#### 2.2.1 Registration of milk composition

The daily fat-to-protein ratio for each cow was recorded using an in-line milk analyzer developed by Brolis Sensor Technology (Vilnius, Lithuania). This device operates within the 2,100–2,400 nm spectral range and utilizes a specially designed GaSb-based, widely tunable external cavity laser spectrometer. It continuously monitored milk flow in transmission mode throughout the milking process. The molecular absorption spectra collected were processed to determine the levels of key milk components. Installed directly on milking stalls or robotic milking systems, this compact “mini spectroscope” required no additional reagents or maintenance. Milk composition was measured every five seconds during every milking, and final values representing the full milking session were calculated by averaging fat and protein levels, weighted by milk flow dynamics.

#### 2.2.2 Registration of rumination time, reticulorumen temperature, water intake, and cow activity

Reticulorumen parameters—including temperature, rumination time, and physical activity—were monitored using SmaXtec boluses (SmaXtec animal care technology^®^, Graz, Austria), designed to support animal health and welfare through continuous, real-time data collection. These boluses also recorded reticulorumen temperature. Following the manufacturer’s instructions, each cow received a single bolus orally, administered by the same experienced veterinarian using a dedicated applicator. The boluses were gravity-settling and designed to remain in the reticulum.

Prior to administration, each bolus was activated, paired with the cow’s unique ear tag for identification, and linked to the central monitoring system. During administration, cows were secured in self-locking head gates, and their heads were gently restrained for bolus placement at the base of the tongue. All cows were monitored for two hours post-administration to check for any adverse reactions.

Data was collected via antennas connected to the SmaXtec system. The system, controlled by a microprocessor and TRR readings using an A/D converter and stored them on an external memory chip. Data compilation was managed through SmaXtec Messenger^®^ software (version 4). Throughout the experiment, we measured reticulorumen temperature, rumination time, cow activity, and water intake.

### 2.3 Groups creation

Holstein dairy cows were eligible for inclusion if they were in early lactation (9 to 59 days in milk), clinically healthy at the start of the study, and housed under uniform feeding and management conditions. Only cows with complete data records from in-line milk analyzers, intraruminal sensors, and repeated blood sampling were included. Cows would have been excluded if they had shown clinical signs of disease—such as mastitis, lameness, displaced abomasum, metritis, or digestive disorders—or if they had incomplete monitoring data. However, no cows were excluded from this study, as all selected animals met the inclusion criteria and remained clinically stable throughout the observation period. The selected group included cows from both first and later lactation. Measurements were taken consistently once a week at the same time each day, resulting in 430 total observations.

Each day between 8:00 and 10:00 a.m., the same veterinarian conducted clinical examinations on the same 27 cows. According to the literature and clinical findings, milk FPR ratios, and blood BHB levels, the cows were categorized into three groups (15):

Low-grade ruminal acidosis (LGRA) group (*n* = 9): Cows in this group showed reduced rumen motility (5–6 contractions per 3 min), milk FPR ratios below 1.2, moderate to severe diarrhea, and undigested feed particles in their feces (confirmed through sieving). They were also assessed for left abomasal displacement by percussion of the left flank. No additional post-calving diseases such as metritis, mastitis, lameness, displaced abomasum, or digestive disorders were detected.

Healthy (H) group (*n* = 9): These cows showed no clinical signs of illness during examinations and remained healthy throughout the whole study period. Their milk fat-to-protein ratios ranged between 1.2 and 1.5, and their blood BHB concentrations were up to 1.2 mmol/L.

Subclinical Ketosis (SCK) group (*n* = 9): Hyperketonemia was identified from samples collected during weekly farm visits, scheduled to coincide with feeding—specifically, two to four hours after the delivery of fresh feed—to capture peak BHB concentrations. During each sampling, cows were secured in a headlock or resting stall, and a small blood sample was drawn from the coccygeal vein using a needle-tipped syringe. These cows had milk FPR above 1.5 and blood BHB concentrations exceeding 1.2 mmol/L, with no visible clinical symptoms after calving. They remained in this group throughout the study.

### 2.4 Statistical analysis

Statistical analysis was conducted using SPSS version 25.0 (SPSS Inc., Chicago, IL, United States). The normality of variable distributions was assessed using descriptive statistics and the Kolmogorov–Smirnov test. Results were presented as means ± standard error of the mean (SEM). Pearson correlation analysis was performed to examine linear relationships between the studied variables. For repeated measurements and comparisons across different days of the experiment, one-way ANOVA and the General Linear Model (GLM) for repeated measures were applied. 3. Results.

Significant differences in physiological and metabolic traits were observed across the different FPR classes (Table 1). Among the most notable findings, cows in the SCK group exhibited significantly lower lactose content compared to the LGRA and H groups (*p* < 0.001), suggesting altered carbohydrate metabolism associated with subclinical ketosis. Similarly, protein content was significantly reduced in the SCK group compared to both the LGRA (*p* < 0.001) and H groups (*p* < 0.001), with a smaller but still significant difference observed between LGRA and H (*p* < 0.05). In contrast, fat content was highest in the SCK group and lowest in the LGRA group (*p* < 0.001), reflecting the influence of energy balance on milk composition.

**Table 1:** Investigated traits according to milk fat to protein ratio class.

Investigated traits	FPR class (M ± SEM)		
	Low-grade ruminal acidosis (LGRA)	Healthy (H)	Subclinical ketosis (SCK)
Lactose	4.78 ± 0.01*** SCK	4.74 ± 0.02*** SCK	4.60 ± 0.03*** LGRA, H
Milk yield, kg	37.74 ± 1.46	36.44 ± 1.14	32.98 ± 1.14
Body condition score (BCS)	3.41 ± 0.04	3.45 ± 0.04	3.18 ± 0.13
Reticulorumen temperature	38.55 ± 0.03	38.61 ± 0.04	38.71 ± 0.03
Rumination (min/24 h)	521.08 ± 6.48*** SCK	514.70 ± 7.11** SCK	458.25 ± 17.55*** LGRA, H
Activity	5.45 ± 0.26** SCK	5.27 ± 0.18*** SCK	7.18 ± 0.55*** LGRA, H
Water intake	132.56 ± 4.61	123.68 ± 4.12	104.43 ± 9.15
Albumin (ALB)	36.25 ± 0.50	35.40 ± 0.34	35.53 ± 0.55
Alkaline phosphatase (ALP)	52.85 ± 2.72	47.66 ± 1.84	45.92 ± 3.89
Alanine aminotransferase (ALT)	27.23 ± 0.87	25.31 ± 0.62	23.68 ± 1.70
Aspartate aminotransferase (AST)	87.27 ± 3.11	84.16 ± 2.21	93.34 ± 6.55
Calcium (Ca)	2.48 ± 0.03	2.47 ± 0.02	2.50 ± 0.05
C-reactive protein (CRP)	9.85 ± 0.71	10.59 ± 0.68	11.19 ± 0.98
Non-esterified fatty acids (NEFA)	0.21 ± 0.02** SCK	0.28 ± 0.02	0.39 ± 0.07** LGRA
Iron (Fe)	21.48 ± 0.70** H, SCK	18.46 ± 0.63** LGRA	16.62 ± 1.54** LGRA
Gamma-Glutamyl Transferase (GGT)	27.89 ± 0.88	29.08 ± 0.82	28.41 ± 2.02
Glucose (GLUC)	3.55 ± 0.05	3.43 ± 0.05	3.53 ± 0.10
Magnesium (Mg)	1.04 ± 0.02	1.03 ± 0.02	0.97 ± 0.06
Triglycerides (TRIG)	0.10 ± 0.01	0.10 ± 0.01	0.10 ± 0.01
UREA	4.16 ± 0.10	4.15 ± 0.11	3.74 ± 0.16

*represent statistically significant differences between F:P ratio FPR class. *p < 0.05, **p < 0.01, ***p < 0.001.

Rumination time was significantly shorter in the SCK group than in both the LGRA and H groups (*p* < 0.001), indicating a potential impact of metabolic stress on feeding behavior. Interestingly, activity levels were highest in the SCK group and significantly greater than those in the LGRA (*p* < 0.01) and H (*p* < 0.001) groups, possibly reflecting increased restlessness.

A clear pattern was also observed in blood metabolites. NEFA concentrations were significantly elevated in the SCK group compared to the LGRA group (*p* < 0.01), consistent with greater lipomobilization during negative energy balance. Serum iron levels were significantly lower in the SCK group compared to LGRA (*p* < 0.01), and a significant difference was also observed between LGRA and H (*p* < 0.01), suggesting alterations in iron metabolism under metabolic stress. Other traits did not show statistically significant differences between FPR classes (*p* > 0.05).

**Figure 1:**
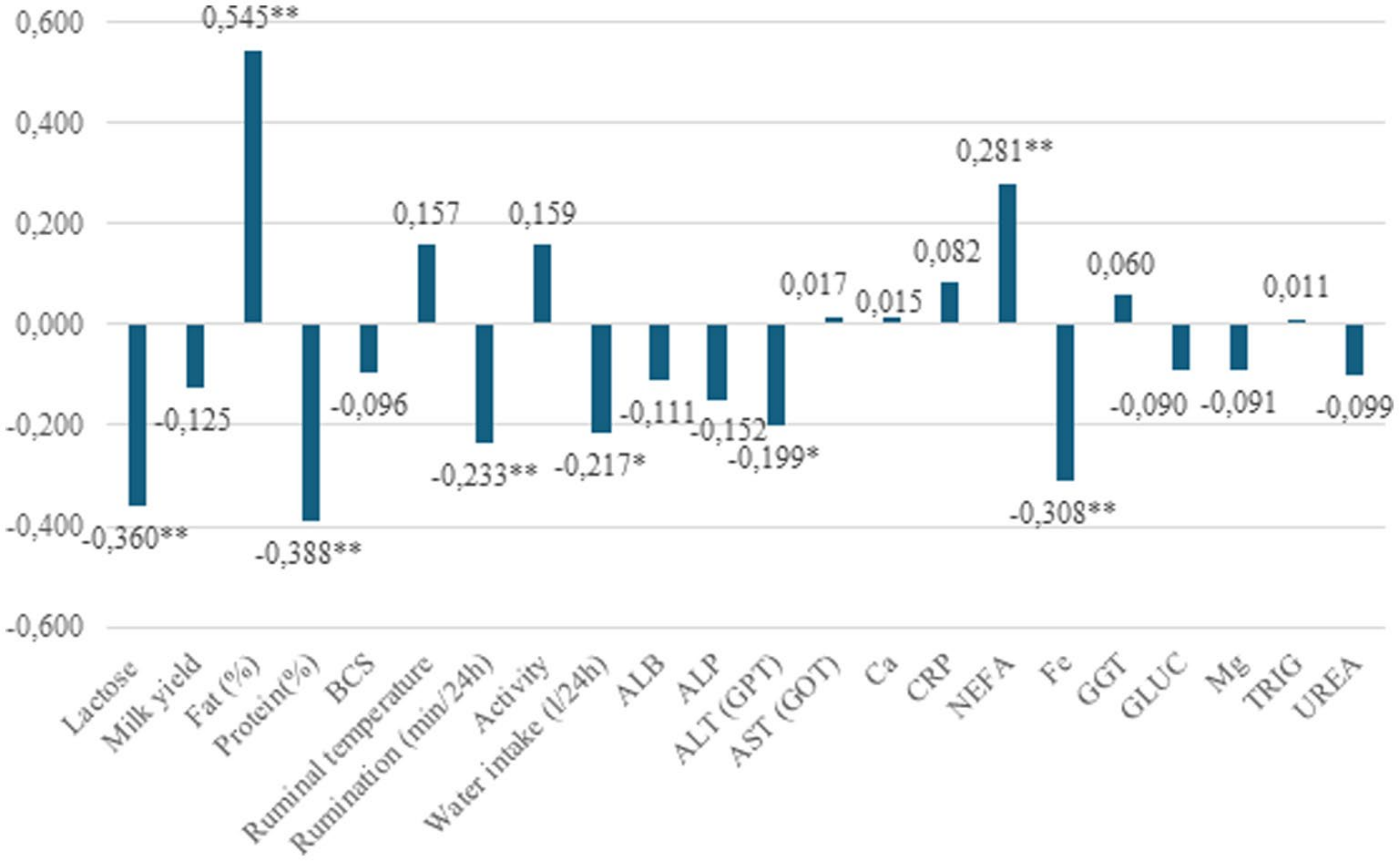
Relationship of FPR class with investigated traits. *P < 0.05, **P < 0.01, ***P < 0.001.

Significant correlations between FPR and selected parameters are summarized in Figure 1. A low but statistically significant positive correlation was detected between FPR and NEFA (r = 0.281, *p* < 0.01). In contrast, negative correlations were found between FPR and lactose (r = −0.360, *p* < 0.01), protein content (r = −0.388, *p* < 0.01), rumination time (r = −0.233, *p* < 0.01), iron (r = −0.308, *p* < 0.01), water intake (r = −0.217, *p* < 0.01), and ALP (GPT) activity (r = −0.199, *p* < 0.05). These consistent negative correlations suggest that as FPR increases, several indicators of metabolic and physiological stability tend to decline.

## 3 Discussion

The analysis of the examined traits across different FPR classes revealed notable differences in this study. When the FPR increased, the lactose content in milk decreased. On average, the lactose concentration was 3.04% higher in the healthy group compared to the subclinical ketosis group, and 3.91% higher in the low-grade ruminal acidosis group than in the subclinical ketosis group. A negative correlation between milk lactose content and FPR was also identified (r = −0.360). This supports the idea that cows with a higher fat-to-protein ratio produce milk with less lactose since it implies that the lactose content tends to decrease as the fat and protein levels in milk increase (13). Other scientists supports this observation, showing that a negative energy balance in the early stages of lactation causes both a decrease in protein levels and an increase in fat production, which impacts the FPR and may be a sign of metabolic disorders (16). Lactose levels and other changes in milk composition are common manifestations of such metabolic changes. Moreover, it has been demonstrated that ketosis adversely affects the levels of lactose and protein in milk. Ketosis increases fat content while decreasing lactose percentage and yield, indicating a change in energy utilization that negatively impacts milk composition (17). Increased fat, which frequently results from ketosis or negative energy balance, can cause decreases in lactose and protein, highlighting the necessity of an integrated approach to dairy cow health management in order to maximize milk composition (18).

Real-time monitoring technologies are also significant for monitoring the health status of dairy cows. Rumination time showed significant differences between groups in our study. Rumination time was 12.32% higher in healthy cows compared to those with subclinical ketosis, and 13.71% higher in the low-grade ruminal acidosis group than in the subclinical ketosis group. Rumination is an essential part of cows’ digestive processes because it helps the rumen ferment efficiently and breaks down complex plant materials mechanically. These results are supported by the negative correlation found between FPR and rumination time (r = −0.233). According to a study by Kaufman et al. (19) rumination time had a positive correlation with milk fat percentage but a negative correlation with milk yield and protein percentage. This result implies that rumination time may decrease as the FPR ratio rises, indicating a comparatively higher fat content relative to protein content. The energy requirements of increased fat synthesis may be the cause of this decline, which would limit the amount of time available for rumination. Another important factor is how rumen fermentation processes affect rumination. Modifications in dietary fat content may cause changes in ruminal fermentation patterns, which may then affect rumination time and milk composition. Because of the change in energy allocation, altered fat fermentation dynamics may result in a reduction in total rumination time while simultaneously increasing energy availability for milk fat production (20). However, cows in the low-grade ruminal acidosis group exhibited the longest rumination time among all groups. This may reflect an early adaptive response to decreasing ruminal pH, where cows increase rumination in an attempt to buffer the rumen environment before clinical signs become apparent (21). These findings underscore the complexity of rumen health regulation, particularly during dietary transitions that can challenge metabolic and digestive stability (22).

Another important parameter recorded in the study through the use of innovative technologies was activity. The highest activity level was detected in the subclinical ketosis group. Activity was 36.24% higher in the subclinical ketosis group compared to the healthy group, and 31.74% higher than in the low-grade ruminal acidosis group. Although higher activity is usually associated with good health and well-being in dairy cows, under conditions of subclinical ketosis it may instead signal a compensatory behavioral reaction to internal metabolic strain (15). In early lactation, reduced feed intake in cows with subclinical ketosis may prompt increased locomotion associated with feed-seeking behavior (23). In this case, elevated activity should not be automatically interpreted as a positive indicator—it may reflect discomfort or imbalance in energy metabolism rather than improved physiological status (12). For this reason, activity data should always be evaluated in combination with other behavioral and physiological parameters, such as changes in rumination or feeding behavior, to help distinguish between normal movement patterns and those driven by metabolic stress (3). An interesting area of research is the connection between increased fat-to-protein ratio and cow activity, especially when considering metabolic health. Certain physiological and behavioral changes in cows, such as changes in activity levels, are correlated with an increase in the FPR (24). Because of the energy imbalances brought on by their condition, cows in subclinical ketosis are more likely to experience other metabolic disorders, even if they may be more active when foraging or looking for food. Due to reduced metabolic efficiency, this may lead to increased activity driven by energy-seeking behavior, rather than reflecting a beneficial or productive physiological response (25). It can be difficult to keep an eye out for activity changes in cows with subclinical ketosis because subtle behavioral changes are harder to spot than in cows with clinical symptoms. By detecting variations in typical activity levels, a greater focus on activity tracking using innovative technologies may help in the early identification of these metabolic problems (26).

Regular blood tests can also help monitor the health status of cows. The blood biochemical analysis revealed that NEFA levels showed significant differences, with the highest value observed in the subclinical ketosis group (0.39 ± 0.07). The average difference in NEFA levels between the low-grade ruminal acidosis and subclinical ketosis groups was 85.71%, with significantly higher concentrations observed in the subclinical ketosis group. Although NEFA concentrations in all cow groups remained within the physiological range, a trend was observed indicating that NEFA levels increased with rising FPR values. An increase in the fat-to-protein ratio or the presence of subclinical ketosis is associated with elevated concentrations of NEFA (27). This is further supported by the positive correlation between FPR and NEFA (r = 0.281) found in our study. Excessive fat mobilization during negative energy balance conditions raises NEFA concentrations, which exacerbates metabolic disorders like ketosis. The necessity of appropriate nutritional management to avoid these imbalances is highlighted by the fact that these problems usually occur during the transition period (28). FPR reflects not only milk composition but also the metabolic processes that drive elevated NEFA levels. According to Antanaitis et al. (9) NEB and adipose tissue mobilization, which is linked to elevated NEFA and ketone bodies, are correlated with increases in the milk FPR. This shows that the metabolic processes that lead to elevated NEFA levels are reflected in the FPR in addition to the composition of milk. This relationship emphasizes how crucial it is to track metabolic markers and milk composition in order to evaluate animal welfare and productivity, particularly during crucial times like the early stages of lactation.

Another statistically significant indicator identified through blood biochemical analysis was iron (Fe). The lowest value of Fe content was estimated in the subclinical ketosis group (16.62 ± 1.54). Fe concentration was 29.23% higher in the low-grade ruminal acidosis group compared to the subclinical ketosis group. In our study, we also found a negative correlation between the FPR and Fe concentration (r = −0.308). Although the observed statistical relationship between FPR and iron concentration is relevant, its biological significance remains unclear. Subclinical ketosis, even in the absence of obvious clinical signs, is known to cause various metabolic disturbances—such as intensified fat mobilization, altered glucose metabolism, and imbalances in nutrient uptake—which could have an impact on mineral levels, including iron (29). It is possible that the metabolic pathways that are triggered during subclinical ketosis, specifically fat mobilization and altered glucose metabolism, will hinder the absorption of minerals, including iron (30). There are also indications that cows with elevated concentrations of NEFA may experience disturbances in nutritional balance, including impaired iron metabolism. Metabolic stress during subclinical ketosis can affect the overall nutritional balance in cows (27). Iron availability may be influenced by a number of factors, including high NEFA concentrations, inadequate nutritional status, and the body’s reaction to metabolic stress. A complex subject in dairy science is the connection between the FPR in cows’ milk and their blood iron levels. Although the findings are promising, the biological significance of reduced iron levels in cows with subclinical ketosis remains unclear. The underlying mechanisms are not fully understood and may be related to reduced feed intake, inflammation, or impaired absorption. Strong evidence for a direct relationship between milk fat-to-protein ratio and blood iron levels is lacking in the current literature. Although metabolic stress may affect mineral balance, the relationship between increased FPR and low Fe levels is still poorly documented. Therefore, these results should be interpreted with caution and further studies are needed to determine whether iron fluctuations are a consistent biomarker of blood iron levels.

This study showed that changes in milk composition, rumination behavior, activity levels, and blood biochemical parameters like NEFA and iron concentrations are all strongly correlated with changes in the fat-to-protein ratio in early-lactation dairy cows. The use of innovative real-time monitoring technologies provided a detailed insight into the metabolic and health status of the cows, highlighting the importance of integrated monitoring systems for early detection of metabolic disorders like subclinical ketosis. However, this research also highlights several areas for improvement and has some significant limitations that should be acknowledged. Future research should think about expanding the sample size to improve the findings’ generalizability and statistical power. For better understanding long-term metabolic trends and recovery patterns, the monitoring period should be extended beyond the early lactation phase. Additionally, using more accurate diagnostic instruments, such as continuous rumen monitoring devices and pH measurements, may help identify subclinical conditions, especially acidosis, earlier and with greater precision. Future research can significantly enhance dairy herd performance and welfare by expanding research approaches and advancing monitoring technologies. In addition, greater attention should be directed toward the dissemination of scientific findings to the broader public. Recent work has demonstrated the potential of social media as an effective channel for science communication in animal agriculture. For example, a study utilizing instagram showed that complex topics—such as the use of biosensing technologies for precision monitoring of dairy cattle health—can be effectively communicated to diverse audiences (31, 32). Such initiatives illustrate how digital platforms not only enhance accessibility but also foster community engagement and counteract misinformation. They also complement the emerging role of influencers in shaping discourse around animal health and dairy production, offering a valuable avenue for extending the reach and impact of evidence-based practices.

## 4 Conclusion

This study underscores the importance of integrating real-time behavioral and milk composition monitoring tools for the early detection of subclinical metabolic disorders in dairy cows. The findings demonstrate a clear association between the FPR in milk and several physiological, behavioral, and metabolic indicators during early lactation. Notable alterations in blood parameters, rumination and activity patterns, and milk composition were observed in cows exhibiting elevated FPR values, reflecting underlying metabolic imbalances.

The application of advanced, non-invasive technologies—such as intra-ruminal boluses and in-line milk analyzers—enabled continuous monitoring of relevant parameters, enhancing both the early identification of health issues and the understanding of their development over time. This approach offers valuable insights into the complex interplay between metabolic health, productivity, and animal welfare, and highlights the potential for precision livestock technologies to support proactive herd management.

Overall, the findings of this study reinforce the applicability of the FPR as a valuable indicator for routine herd health monitoring and management. The integration of milk composition data with behavioral observations and blood-based biomarkers enhances the potential to identify subclinical metabolic disturbances, thereby supporting more proactive and informed decision-making by dairy producers and veterinarians.

## Data Availability

The original contributions presented in the study are included in the article/supplementary material, further inquiries can be directed to the corresponding author.

## References

[1] Akbar MO Shahbaz Khan MS Ali MJ Hussain A Qaiser G Pasha M. IoT for development of smart dairy farming. J Food Qual. (2020) 2020:1–8. doi: 10.1155/2020/4242805, PMID: 40547809

[2] Lamanna M Bovo M Cavallini D. Wearable collar technologies for dairy cows: a systematized review of the current applications and future innovations in precision livestock farming. Animals. (2025) 15:458. doi: 10.3390/ani15030458, PMID: 39943229 PMC11815998

[3] Młynek K Górka P Rzewuska K Szumacher-Strabel M. Early diagnosis of subclinical ketosis and ruminal acidosis using in-line milk analysis and cow behavior data. Front Anim Sci. (2025) 4:1547395. doi: 10.3389/fanim.2025.1547395

[4] Mammi LME Cavallini D Fustini M Fusaro I Giammarco M Formigoni A. Calving difficulty influences rumination time and inflammatory profile in Holstein dairy cows. J Dairy Sci. (2020) 103:4890–9. doi: 10.3168/jds.2020-18867 33131814

[5] Antanaitis R Džermeikaitė K Krištolaitytė J Girdauskaitė A Arlauskaitė S Tolkačiovaitė K. The relation between milk lactose concentration and the rumination, feeding, and locomotion behavior of early-lactation dairy cows. Animals (Basel). (2024) 14:836. doi: 10.3390/ani14060836, PMID: 38539934 PMC10967315

[6] Bobbo T Fiore E Gianesella M Morgante M Gallo L Ruegg PL. Variation in blood serum proteins and association with somatic cell count in dairy cattle from multi-breed herds. Animal. (2017) 11:2309–19. doi: 10.1017/S1751731117001227, PMID: 28560948

[7] Magro S Costa A Cavallini D Chiarin E De Marchi M. Phenotypic variation of dairy cows’ hematic metabolites and feasibility of non-invasive monitoring of the metabolic status in the transition period. Front Vet Sci. (2024) 11:1437352. doi: 10.3389/fvets.2024.1437352, PMID: 39654842 PMC11626799

[8] Schcolnik T. Using milk fat-to-protein ratio to evaluate dairy cows energy balance status. J Anim Sci. (2016) 94:54–5. doi: 10.2527/jas2016.94supplement5.54

[9] Guliński P. Ketone bodies – causes and effects of their increased presence in cows’ body fluids: a review. Vet World. (2021) 14:1492–503. doi: 10.14202/vetworld.2021.1492-1503, PMID: 34316197 PMC8304442

[10] Antanaitis R Džermeikaitė K Januškevičius V Šimonytė I Baumgartner W. In-line registered milk fat-to-protein ratio for the assessment of metabolic status in dairy cows. Animals (Basel). (2023) 13:3293. doi: 10.3390/ani13203293, PMID: 37894017 PMC10603915

[11] Vlček MJ Kasarda R. Changes of fat-to-protein ratio from start to the mid-lactation and the impact on milk yield. J Cent Eur Agric. (2016) 17:1194–203. doi: 10.5513/JCEA01/17.4.1830

[12] Hajek F Mansfeld R. Übersicht zu Zusammenhängen zwischen Änderungen der Bewegungsintensität und Ketose bei Milchkühen. Tierarztl Prax Ausg G Grosstiere Nutztiere. (2019) 47:380–9. doi: 10.1055/a-1037-9173 31810085

[13] Sneddon NW López-Villalobos N Davis SR Re H Shalloo L. Genetic parameters for milk components including lactose from test day records in the New Zealand dairy herd. N Z J Agric Res. (2014) 58:97–107. doi: 10.1080/00288233.2014.978482

[14] Plaizier JC Ametaj BN Khafipour E. Evidence of systemic inflammation in dairy cows with subacute ruminal acidosis. Animals. (2022) 12:2129. doi: 10.3390/ani12162129 36009720 PMC9404850

[15] Antanaitis R Džermeikaitė K Krištolaitytė J Ribelytė I Bespalovaitė A Bulvičiūtė D. Alterations in rumination, eating, drinking and locomotion behavior in dairy cows affected by subclinical ketosis and subclinical acidosis. Animals (Basel). (2024) 14:384. doi: 10.3390/ani14030384, PMID: 38338027 PMC10854656

[16] Gantner V Bobić T Potočnik K. Prevalence of metabolic disorders and effect on subsequent daily milk quantity and quality in Holstein cows. Arch Anim Breed. (2016) 59:381–6. doi: 10.5194/aab-59-381-2016

[17] Yang W Zhang B Xu C Zhang H Xia C. Effects of ketosis in dairy cows on blood biochemical parameters, milk yield and composition, and digestive capacity. J Vet Res. (2019) 63:555–60. doi: 10.2478/jvetres-2019-0059, PMID: 31934667 PMC6950442

[18] Televičius M Juozaitienė V Malašauskienė D Antanaitis R Rutkauskas A Urbutis M. Inline milk lactose concentration as biomarker of the health status and reproductive success in dairy cows. Agriculture. (2021) 11:38. doi: 10.3390/agriculture11010038

[19] Tricárico JM Johnston JD Dawson KA Hanson-Dorr KC McLeod KR Harmon DL. The effects of an aspergillus oryzae extract containing alpha-amylase activity on ruminal fermentation and milk production in lactating Holstein cows. Anim Sci. (2005) 81:365–74. doi: 10.1079/asc50410365

[20] Kaufman EI Asselstine V LeBlanc S Duffield T DeVries T. Association of rumination time and health status with milk yield and composition in early-lactation dairy cows. J Dairy Sci. (2018) 101:462–71. doi: 10.3168/jds.2017-12909, PMID: 29055534

[21] Gao X Oba M. Relationship of severity of subacute ruminal acidosis to rumen fermentation, chewing activities, sorting behavior, and milk production in lactating dairy cows fed a high-grain diet. J Dairy Sci. (2014) 97:3006–16. doi: 10.3168/jds.2013-7472, PMID: 24612805

[22] Zhao C Liu G Li X Guan Y Wang Y Xue Y. Inflammatory mechanism of rumenitis in dairy cows with subacute ruminal acidosis. BMC Vet Res. (2018) 14:135. doi: 10.1186/s12917-018-1463-7 29673406 PMC5909223

[23] Pérez-Báez J Risco C Chebel R Gomes G Greco L Tao S. Association of dry matter intake and energy balance prepartum and postpartum with health disorders postpartum: part II. Ketosis and clinical mastitis. J Dairy Sci. (2019) 102:9151–64. doi: 10.3168/jds.2018-15879, PMID: 31326169

[24] Zhang Z Liu G Li X Gao L Guo C Wang H. Evaluation of the change of serum copper and zinc concentrations of dairy cows with subclinical ketosis. Biol Trace Elem Res. (2010) 138:8–12. doi: 10.1007/s12011-009-8606-4, PMID: 20101474

[25] Venjakob P Staufenbiel R Heuwieser W Borchardt S. Association between serum calcium dynamics around parturition and common postpartum diseases in dairy cows. J Dairy Sci. (2021) 104:2243–53. doi: 10.3168/jds.2019-17821, PMID: 33246622

[26] Rodríguez E Arís A Bach À. Associations between subclinical hypocalcemia and postparturient diseases in dairy cows. J Dairy Sci. (2017) 100:7427–34. doi: 10.3168/jds.2016-12210 28690056

[27] Ayvazoğlu C Gökçe E. Investigation of the prevalence of ketosis in cows in Ardahan region in Turkey. Kocatepe Vet J. (2020) 13:406–12. doi: 10.30607/kvj.798027

[28] Wang J Zhu X She G Kong Y Guo Y Wang Z. Serum hepatokines in dairy cows: periparturient variation and changes in energy-related metabolic disorders. BMC Vet Res. (2018) 14:236. doi: 10.1186/s12917-018-1560-7, PMID: 30103741 PMC6090689

[29] González F Muíño R Pereira V Gaona RC Benedito JL. Relationship among blood indicators of lipomobilization and hepatic function during early lactation in high-yielding dairy cows. J Vet Sci. (2011) 12:251–5. doi: 10.4142/jvs.2011.12.3.251 21897097 PMC3165154

[30] Sun L Zhang H Wu L Shu S Xia C Xu C. 1H-nuclear magnetic resonance-based plasma metabolic profiling of dairy cows with clinical and subclinical ketosis. J Dairy Sci. (2014) 97:1552–62. doi: 10.3168/jds.2013-6757, PMID: 24440255

[31] Erickson N Neave HW Weary DM. Using Instagram to communicate about precision monitoring technologies in dairy cattle. J Dairy Sci. (2024) 107:25347. doi: 10.3168/jds.2024-25347

[32] Čobanović K Pajić M Radinović M Simonović M Csorba C Kučević D. Environmental factors affecting milk composition in Holstein cattle breed. J Hell Vet Med Soc. (2022) 72:3355. doi: 10.12681/jhvms.29374

